# Association of the existence of CRISPR-Cas system and antimicrobial resistance in multi-drug resistant *Klebsiella pneumoniae* in Egypt

**DOI:** 10.1038/s41598-025-26706-6

**Published:** 2025-11-25

**Authors:** Nagwan Galal El Menofy, Aya Nassef Payoumi, Mohamed A. Eissa, Amany El-Sharif

**Affiliations:** 1 Microbiology and Immunology Department, Faculty of Pharmacy, Al- Azhar University, Cairo, 11765 Egypt; 2 Biotechnology Department, Faculty of Agriculture, Al-Azhar University, Cairo, 11754 Egypt; 3 PanAfrican University, Yaoundé, Cameroon

**Keywords:** CRISPR/Cas, *K. pneumoniae*, Multidrug resistant, Carbapenem resistance, Colistin, Association, Diseases, Microbiology, Molecular biology

## Abstract

The CRISPR-Cas systems are supposed to be associated with antibiotic susceptibility. *Klebsiella pneumoniae* (*K. pneumoniae*) is a major multidrug-resistant (MDR) pathogen that may cause severe infections. This study aimed to detect the antimicrobial resistance (AMR) of *K. pneumoniae* isolates in addition to determine the association between the existence of CRISPR-Cas systems and the presence of AMR in Egypt. The antibiotic susceptibility patterns of 100 *K. pneumoniae* isolates were determined using the Kirby Bauer disc diffusion and broth microdilution methods. The frequency of carbapenem resistance encoding genes (*bla*_*KPC*_, *bla*_*OXA*_, *bla*_*IMP*,_ *bla*_*NDM*_, and *bla*_*VIM*_), ESBLs encoding genes *(bla*_*TEM*_), aminoglycoside resistance encoding genes *(aac(3)-Ia*,* aac(3)-IIa*, colistin resistance encoding genes **(***mcr-1*, *mcr-2)* and tetracycline resistance encoding genes (*tetB*) were determined using polymerase chain reaction (PCR). The presence or absence of CRISPR-Cas systems was determined by detection of Cas genes (Cas1 or Cas3) in conjunction with one of CRISPR arrays 1, 2 or 3. Kirby Bauer disc diffusion revealed that 95% of isolates were MDR. The resistance rates of *K. pneumoniae* isolates to amikacin, meropenem, and colistin were 76%, 67%, and 41% respectively by broth microdilution assay. Among selected 41 *K. pneumoniae*, the frequency of ESBLs; *bla*_*TEM*_ was 92.7%, while the frequency of *bla*_*NDM*_ *bla*_*OXA*_ *bla*_*VIM*_ *bla*_*IMP*_ and *bla*_*KPC*_ was 95.1%, 95.1%, 39%, 19.5% and 14.6% respectively. The frequency of *mcr-1* and *mcr-2* was 70.7% and 65.9%. Additionally, the frequency of *aac(3)-Ia* was 12.2%, and *aac(3)-IIa* was 87.8%, while the frequency of *tetB* was 100%. Our isolates exhibited varied profiles for CRISPR-Cas systems, where 65.9% were positive for CRISPR-Cas system. No general significant positive correlation between AMR and the presence ofCRISPR-Cas system was detected; however, a significant difference is present for imipenem, colistin and chloramphenicol phenotypic resistance andfor aac(3)-IIa and mcr-1 genes (*P* value > 0.1). A significant positive correlation was detected between AMR for imipenem, colistin and chloramphenicol and for aac(3)-IIa and mcr-1 genes and thepresence of CRISPR-Cas system.

**Supplementary Information:**

The online version contains supplementary material available at 10.1038/s41598-025-26706-6.

## Introduction

The clustered regularly interspaced short palindromic repeats (CRISPRs) and CRISPR-associated (Cas) genes/proteins, are part of the adaptive immune system in prokaryotes and found in roughly 50% of bacterial genomes and 87% of archaea^1^. CRISPR-Cas adaptive immune systems are one of the most prevalent defenses in bacteria but their role in limiting the spread of antimicrobial resistance genes (ARGs) is not well-understood^2^.

Makarova and colleagues in 2011 introduced a system for categorizing CRISPR-Cas systems into three primary types: type I (identified by the cas3 gene), type II (characterized by the cas9 gene), and type III (distinguished by the cas10 gene), which has since become the standard terminology in the field^3^. Cas enzymes utilize CRISPR sequences to identify and cleave the complementary strands of target DNA. CRISPR arrays enable bacteria or archaea to retain information about previous viral encounters, with viral DNA copies being integrated into their genomes as “spacers” between the short DNA repeats of CRISPR arrays when encountering virus^4^.

Several studies have established a correlation between CRISPR-Cas systems and increased susceptibility to antibiotics, as well as reduced plasmid acquisition in various bacterial species. Some research also suggests that these systems may play a role in regulating bacterial pathogenicity^5^. For instance, the CRISPR-Cas system influences the contents of prophages in *Streptococcus pyogenes*, impacting its virulence^6^. Additionally, Mackow et al. found that *Klebsiella pneumoniae* with CRISPR-Cas systems exhibited heightened sensitivity to carbapenems^7^.

Conversely, some studies indicate that bacteria can evolve mechanisms to bypass the CRISPR-Cas system’s effects, such as acquiring mutations that disable its function^8,9^. Therefore, there is significant potential for further research into the impact of CRISPR-Cas systems on the virulence and antibiotic resistance of various bacterial pathogens.

Antimicrobial resistance carries significant social and economic repercussions, leading to prolonged sickness, extended hospitalization, and the requirement for more expensive medications, with a disproportionate impact on lower- and middle-income nations^10^. The primary catalysts behind the rise of MDR in bacteria stem from the inappropriate and excessive use of antibiotics^11^. Many microbial elements contribute to the development of AMR. Microbial factors such as genomic plasticity, genetic exchanges (e.g., horizontal genetic transfer [HGT]), mutations, enzyme modifications, degradative enzymes, changes in pathways, and activation of efflux pumps play a crucial role in the emergence and rise of AMR^12^. A pan-genome refers to a collection of genes that collectively make up a certain bacterial species^13^. A pan-genome is divided into three sections: the core (persistent) genome, which is shared by at least 95% of bacterial strains, the shell (accessory) genome, which is shared by at least 15% but less than 95% of strains, and the cloud (strain-specific/unique/singleton) genes, which are present in less than 15% of bacterial strains^13,14^. According to genomic studies, *K. pneumoniae’s* pan-genome is around five to six Mbp in size, with five to six kilogenes to be encoded. Of these encodable genes, around seventeen hundred are classified as core genes. However, the remaining genetic pool is the accessory genome which differs between Klebsiella species^15^.

Currently, *K. pneumoniae* has demonstrated resistance to major effective antibiotic categories like carbapenems, cephalosporins, and aminoglycosides, complicating antibiotic treatments and resulting in treatment failures^16^. Several AMR genes, such as *bla*_*KPC*_, *bla*_*OXA−48*_, and *bla*_*NDM−1*_, were initially identified in *K. pneumoniae* before dissemination to other pathogens^17^. Multidrug-resistant bacteria evade the effects of most antibiotics through successive genetic changes and the transfer of mobile genetic elements, highlighting the urgent necessity to investigate resistance development and prevention strategies^18^. The CRISPR-Cas system has been proposed as a novel tool to tackle antibiotic resistance, presenting a promising avenue for developing next-generation antimicrobials to address infectious diseases^19,20^.

In the current study, we aimed to detect the prevalence of AMR detected phenotypically and genotypically and CRISPR-Cas encoding genes in MDR *K. pneumoniae* clinical isolates and analyze the correlation between the presence of CRISPR-Cas system and prevalence of antimicrobial resistance to different antibiotics as of β-lactam, aminoglycoside, tetracycline and colistin particularly addressing a research gap in the Middle Eastern context regarding this association.

## Methods

### Study design and identification of bacterial isolates

All experiments were carried out at the research lab of the Microbiology & Immunology Department, Faculty of Pharmacy, Al-Azhar University, Cairo, Egypt, and the Genomics Lab at Biotechnology Department, Faculty of Agriculture, Al-Azhar University, Cairo, Egypt, during the period from October 2020 to June 2023. A total number of 100 clinical isolates of *K. pneumoniae* isolated from different clinical specimens (presented in supplementary file in Fig.S1) were obtained from the Microbiology lab at Al-Demerdash Hospital, Ain Shams University, Cairo-Egypt.

*K. pneumoniae* isolates were identified biochemically using standard microbiological tests as growth on triple sugar iron agar, Simmon’s citrate agar, indole test, urease tests^21^. etc. Identification of *K. pneumonia* was confirmed using the Vitek-2 automated system (Biomérieux, Marcy-LÉtoile, Paris, France) following the manufacturer’s instructions. All *K. pneumoniae* isolates were preserved at − 20 °C in brain–heart infusion broth with 20% glycerol (Oxoid, London, UK) until use^22^.

### Antimicrobial susceptibility testing using Kirby–Bauer disc diffusion method

The Kirby–Bauer disc diffusion method was used to detect the antibiotic susceptibility of all identified *K. pneumoniae* isolates on Muller–Hinton agar (LAB M Limited, UK) according to the Clinical and Laboratory Standards Institute (CLSI 2023)^23^. All antibiotic discs were supplied by Oxoid, London, UK and included different categories amoxicillin/clavulanic acid (20/10 µg), piperacillin/tazobactam (100/10 µg) [penicillins and beta-lactamase inhibitors], ceftriaxone (30 µg), ceftazidime (30 µg), cefuroxime (30 µg), cefoxitin(30 µg) [cephalosporins], imipenem(10 µg), meropenem (10 µg) [carbapenem], ciprofloxacin (5 µg) [fluoroquinolones], gentamicin (10 µg), amikacin (30 µg) [aminoglycosides], chloramphenicol (30 µg)[glycols], and colistin (10 µg) [polymyxin]^24^. *K pneumoniae* ATCC700603 was used as positive control. The results of the disc diffusion assay were interpreted according to CLSI guidelines^23^. MDR *K. pneumoniae* isolates were identified as non-susceptibility to at least one agent in three antimicrobial classes^25^.

### Antimicrobial susceptibility testing using broth microdilution assay

The MIC for amikacin, meropenem, and colistin was determined using the broth microdilution assay according to the guidelines of the CLSI 2023^23^. Briefly, a 96-well microtiter plate was filled with 100 µL of double-strength MHB medium (LAB M Limited, UK). Then 100 µL of working antibiotic solution was added in each well of the first row of the microtiter plate. Two-fold serial dilution (from 128 to 0.5 µg/mL) was performed by transferring 100 µL of antibiotic from the first well to the subsequent wells of the plate. Fresh bacterial colonies were adjusted to a turbidity equivalent of 0.5 McFarland standard, and each well was inoculated with 7 µL of fresh *K. pneumoniae* inoculum (1.5 × 10^8^) to reach final inoculum density of 10^5^ CFU ml^− 1^ per wells^26^. The plate was incubated for 24 h at 37 °C. After incubation, the MIC was determined as the lowest concentration of antibiotic which showed no visible growth. The results of amikacin and meropenem were interpreted according to the guidelines of CLSI, the breakpoints for AMC were (Sensitive(S) ≤ 16 µg/mL, Intermediate (I) = 32, and Resistant (R) ≥ 64 µg/mL), and for MEM were (S ≤ 1 µg/mL, I = 2, *R* ≥ 4 µg/mL), while the results of colistin were interpreted according to the European Committee on Antimicrobial Susceptibility Testing^27^. The breakpoints for COL were (S ≤ 2 µg/mL, and *R* > 2 µg/mL).

### DNA extraction and primer design

Total genomic DNA was extracted using G-spinTM Total DNA Extraction Kit, (Intron Biotechnology, Keonggi-do, South Korea) according to the manufacturer’s instructions. The DNA was stored at − 20 °C. Primers for ESBLs encoding genes; *bla*_*TEM*_ (the most commonly found in community and livestock environments)^28^, carbapenem resistance encoding genes (*bla*_*KPC*_, *bla*_*OXA*_, *bla*_*IMP*_, *bla*_*NDM*_ *and bla*_*VIM*_), aminoglycosides resistance encoding genes (*aac(3)-Ia* and *aac(3)-IIa*), colistin resistance encoding genes (*mcr-1 and mcr-2*) and tetracycline resistance encoding genes (*tetB*), were designed in the current study according to conserved region of the *Klebsiella pneumoniae* subsp. pneumoniae HS11286. The primers specificity was checked using Primer BLAST tools in NCBI https://www.ncbi.nlm.nih.gov/tools/primer-blast/.

The CRISPR-Cas system encoding genes including *CRISPR1*, *CRISPR2*, *CRISPR3*, *Cas1*, and *Cas3* genes were chosen using the previously published specific primers^29^. All primers are listed in Table 1.

**Table 1:** Sequences of PCR oligonucleotide primers of antimicrobial resistance encoding genes and of CRISPER-CAS system encoding genes.

Gene	Primer sequence 5’ - 3’	Amplicon size (bp)
I-Antimicrobial resistance encoding genes primers		
**ESBLs encoding genes**		
*bla*_*TEM*_	F: TGC GGT ATT ATC CCG TGT TG	297
	R: TCG TCG TTT GGT ATG GCT TC	
**Carbapenem resistance encoding genes**		
*bla*_*KPC*_	F: CGT CTA GTT CTG CTG TCT TG	798
	R: CTT GTC ATC CTT GTT AGG CG	
*bla*_*OXA−48*_	F: GCG TGG TTA AGG ATG AAC AC	438
	R: CAT CAA GTT CAA CCC AAC CG	
*bla*_*IMP*_	F: CTA CCG CAG CAG AGT CTT TGC	589
	R: ACA ACC AGT TTT GCC TTA CC	
*bla*_*NDM*_	F: GCA GCT TGT CGG CCA TGC GGG C	782
	R: GGT CGC GAA GCT GAG CAC CGC AT	
*bla*_*VIM*_	F: AAA GTT ATG CCG CAC TCA CC	865
	R: TGC AAC TTC ATG TTA TGC CG	
**Colistin resistance encoding genes**		
*mcr-1*	F: CGG TCA GTC CGT TTG TTC	309
	R: CTT GGT CGG TCT GTA GGG	
*mcr-2*	F: TGT TGC TTG TGC CGA TTG GA	567
	R: AGA TGG TAT TGT TGG TTG CTG	
**Aminoglycoside resistance encoding genes**		
*aac(3)-Ia*	F: ACC TAC TCC CAA CAT CAG CC	155
	R: ATC TCA CTA CGC GCC TG	
*aac(3)-IIa*	F: CTC TTG ATG GTG CAT GCC TC	247
	R: ATT GAT TCA GCA GGC CGA AC	
**Tetracycline resistance encoding genes**		
*tetB*	F: CAG TGC TGT TGT TGT CAT TAA	571
	R: GCT TGG AAT ACT GAG TGT AA	
**II- CRISPER-CAS system encoding genes primers**^29^.		
*CRISPR1*	F: CAG TTC CTG CAA CCT GGC CT	208
	R: CTG GCA GCA GGT GAT ACA GC	
*CRISPR2*	F: GTA GCG AAA CCC TGA TCA AGC G	620
	R: GCG CTA CGT TCT GGG GAT G	
*CRISPR3*	F: GAC GCT GGT GCG ATT CTT GAG	1888
	R: CGC AGT ATT CCT CAA CCG CCT	
*Cas1*	F: GCT GTT TGT CAA AGT TAC	1173
	R: GGT TTT GAT CGC CTC ATG AGT	
*Cas3*	F: TGG CCG ACA TTT GAT TCA GC	1598
	R: CCA TGC TTA ACA TTC ATC AC	

### Molecular detection of resistance genes and CRISPER-CAS system by the polymerase chain reaction

PCR reaction mixture was performed in volume of 25 µL, including 10 µL of PCR master mix (Williford, Nottinghamshire NG, England), 1 µL of each specific primer (25 nanomoles), 5 µL (250 ng) of DNA template and 8 µL of RNAse free distilled water^30^. The timetable and thermal schedule for each gene are presented in Supplementary Table 1. The amplified products were run on 1% agarose gel (Genetix Biotech, New Delhi, India) stained with ethidium bromide (Bioshop, Ontario, Canada) and photographed under UV illumination. A 100–1000 base-pairs standard DNA ladder (Bengaluru, Karnataka, India) was used for sizing the PCR products^31^.

### Statistical analysis

**Fig. 1:**
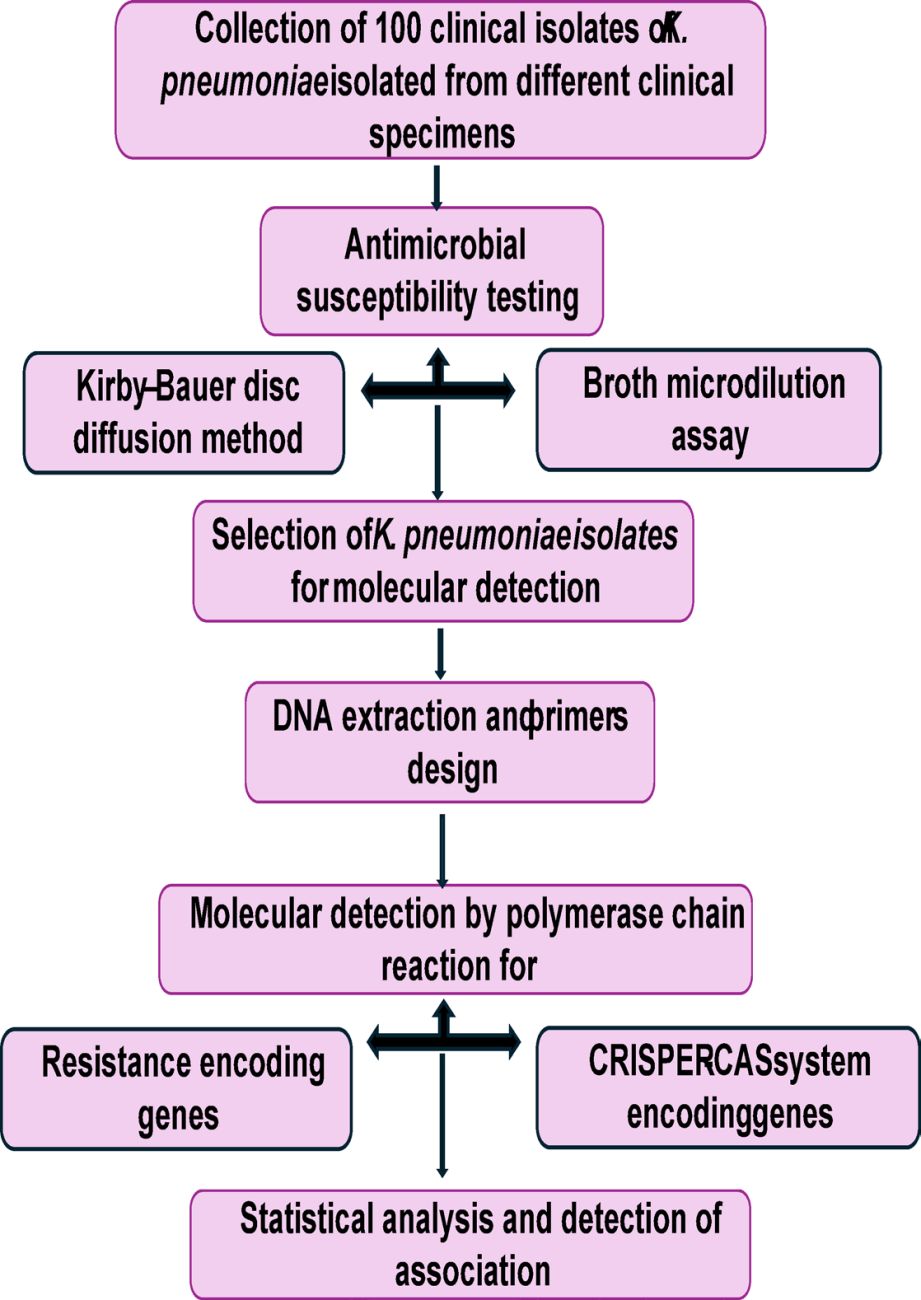
Flow chart showing study design and methodology.

Statistical analysis was performed using IBM SPSS^®^ Statistics version 26 (IBM^®^ Corp., Armonk, NY, USA). Qualitative data were expressed as frequency and percentage. Pearson’s Chi-square test or Fisher’s exact test was used to examine the relation between qualitative variables. All tests were two-tailed. A *p*-value < 0.05 was considered significant. Flow chart that clarifies all performed methods is presented in Fig. 1.

## Results

### Results of antimicrobial susceptibility using Kirby–Bauer disc diffusion method

**Fig. 2:**
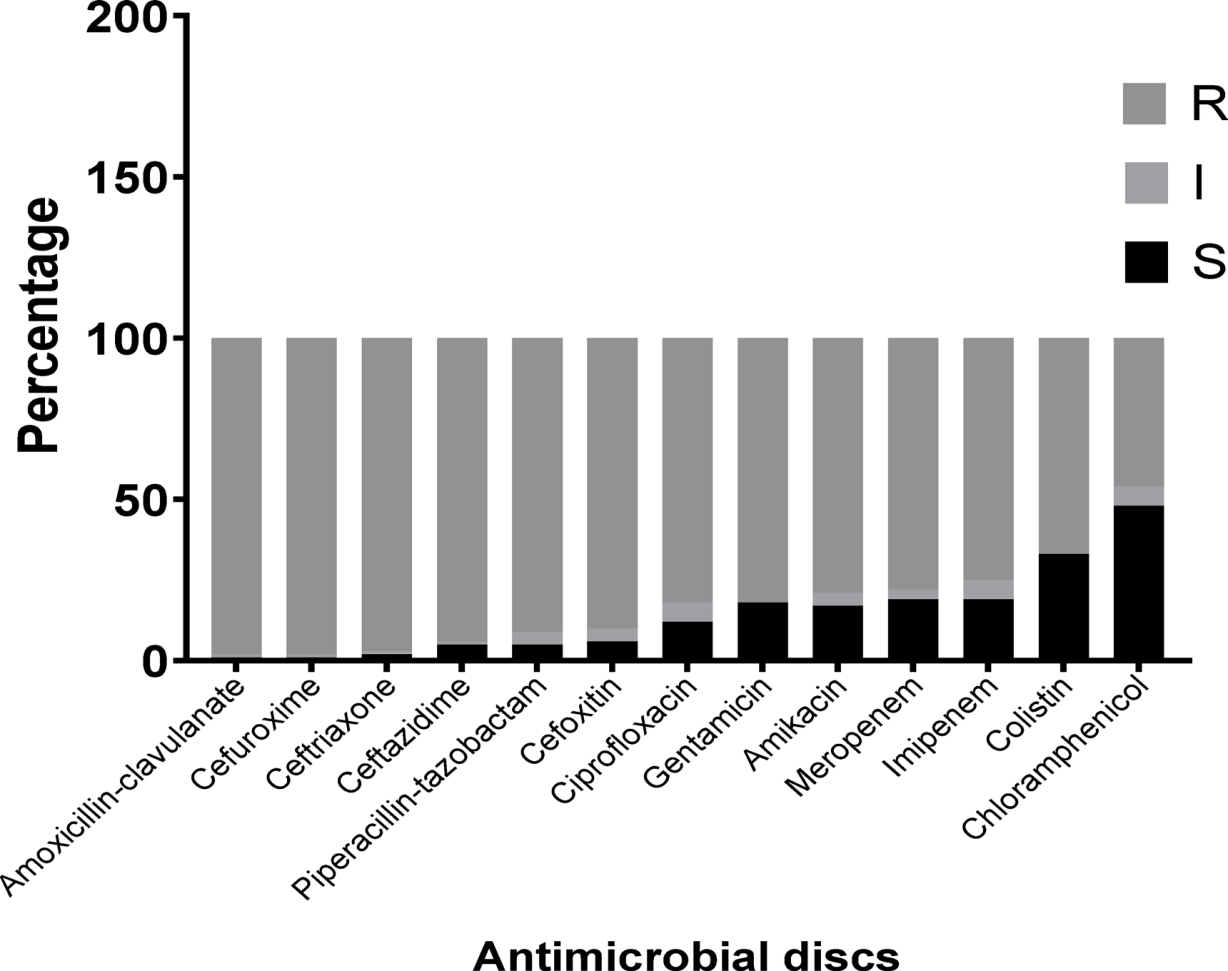
The frequency of antimicrobial resistance of *k. pneumoniae* isolates by Kirby–Bauer disc diffusion method.

Regarding individual antibiotic susceptibility testing, 98% of isolates were resistant to amoxicillin/clavulanic acid and cefuroxime, while 97% of isolates were resistant to ceftriaxone followed by ceftazidime (94%), piperacillin-tazobactam (91%), and cefoxitin (90%). Also, 82% of *K pneumoniae* isolates were resistant to ciprofloxacin and gentamicin. The resistance rate to meropenem, imipenem, and colistin were 78%, 75%, and 67% respectively. On the other hand, only 46% of *K. pneumoniae* isolates showed resistance to chloramphenicol as shown in Fig. 2 and Supplementary Table 2. The results of the Kirby–Bauer disc diffusion method revealed that 95% of *k. pneumoniae* isolates were MDR.

### Results of antimicrobial susceptibility using broth microdilution assay

The MIC results of amikacin, meropenem, and colistin for the 100 *k. pneumoniae* isolates are shown in (Table 2). The resistance rates to amikacin, meropenem and colistin were 76%, 67%, and 41% respectively while 8% and 2% of isolates have intermediate sensitivity to meropenem and amikacin, respectively.

**Table 2:** MIC values of amikacin, meropenem, and colistin by microdilution method.

Antibiotic MIC Range (µg/mL)	Amikacin No (%)	Meropenem No (%)	Colistin No (%)
≥ 64	76 (76%)	22 (22%)	16 (16%)
32	2 (2%)	6 (6%)	2 (2%)
16	2 (2%)	15 (15%)	5 (5%)
8	6 (6%)	8 (8%)	6 (6%)
4	4 (4%)	16 (16%)	12 (12%)
2	5 (5%)	8 (8%)	30 (30%)
1	2 (2%)	7 (7%)	17 (17%)
≤ 0.5	3 (3%)	18 (18%)	12 (12%)
Resistance (%)	76 (76%)	67 (67%)	41 (41%)

### Molecular detection of antimicrobial resistance encoding genes via PCR

Forty-one isolates were selected for molecular identification including 18 isolates that showed MDR profile by Kirby–Bauer disc diffusion method and broth microdilution assay, 5 isolates that were sensitive to amikacin, meropenem, and colistin by Kirby–Bauer disc diffusion method, 6 isolates were sensitive to 2 antibiotics by broth microdilution assay, 7 isolates were sensitive to only 1 antibiotic by broth microdilution assay and five isolates were sensitive to most antibiotic groups.

The analysis of resistance encoding genes revealed a high prevalence of ESBLs, bla_*TEM*_ gene which was detected in 92.7% isolates. Furthermore, genes conferring carbapenem resistance, specifically *the bla*_*NDM*_ *and bla*_*OXA*_, were predominant, identified in 95.1% of isolates. Other carbapenemase genes, including *bla*_*VIM*_, *bla*_*IMP*_ *and bla*_*KPC*_ were present in 39%, 19.5%, and 14.6% of the total isolates, respectively. Regarding colistin resistance, the *mcr-1* and *mcr-2* genes were observed in 70.7% and 65.9% of isolates, respectively. The prevalence of aminoglycoside resistance-encoding genes, *aac(3)-Ia* and *aac(3)-IIa*, was 12.2% and 87.8%, respectively. Notably, the tetracycline resistance-encoding gene *tetB* was ubiquitously detected across all isolates. All PCR results are presented in Table 3 and all PCR gel pictures are presented in supplementary file (figures S2-S17).

**Table 3:** Molecular detection of resistance encoding genes among *K. pneumoniae* isolates.

Gene	Positive isolates No. (%)
**ESBLs encoding genes**	
*bla*_*TEM*_	38(92.7)
**Carbapenem resistance encoding genes**	
*bla*_*NDM*_	39(95.1)
*bla*_*OXA*_	39(95.1)
*bla*_*VIM*_	16(39)
*bla*_*IMP*_	8(19.5)
*bla*_*KPC*_	6(14.6)
**Colistin resistance encoding genes**	
*mcr-1*	29(70.7)
*mcr-2*	27(65.9)
**Aminoglycoside resistance encoding genes**	
*aac(3)-Ia*	5(12.2)
*aac(3)-IIa*	35 (87.8)
**Tetracycline resistance encoding genes**	
*tetB*	41(100

Genotypic profiles of AMR genes among *K. pneumoniae* isolates are presented in Table 4 and revealed that *bla*_*OXA*,_ *bla*_*TEM*_, *bla*_*VIM*_, *mcr-1*,* mcr-2*,* aac(3)-IIa- tetB* genetic profile was harbored by 21.9% of *K. pneumoniae* isolates as shown in Table 4.

**Table 4:** Genotypic profiles of AMR encoding genes among *K. pneumoniae* isolates.

Resistance pattern	Genotypic Profile	Frequency No. (%)
I	*bla*_*OXA−*_*-bla*_*TEM*_*- bla*_*VIM*_*-* *mcr-1- mcr-2- aac(3)-IIa - tetB*	9(21.9) %
II	*bla*_*NDM*_*- bla*_*OXA−*_ *bla*_*TEM−*_ *mcr-1- aac(3)-IIa- tetB*	3(7.3) %
III	*bla*_*NDM*_*- bla*_*OXA−*_ *bla*_*TEM−*_ *bla*_*VIM*_ *- bla*_*KPC−*_ *mcr-1- mcr-2- aac(3)-IIa- tetB*	2(4.9) %
IV	*bla*_*NDM*_*- bla*_*OXA−*_ *bla*_*TEM−*_ *bla*_*VIM*_ *- mcr-1- mcr-2- aac(3)-IIa- tetB*	2(4.9) %
V	*bla*_*NDM*_*- bla*_*OXA−*_ *bla*_*TEM−*_ *- bla*_*IMP−*_ *mcr-1- mcr-2- aac(3)-IIa- tetB*	2 (4.9) %
VI	*bla*_*NDM*_*- bla*_*OXA−*_ *bla*_*TEM−*_ *bla*_*VIM−*_*- mcr-1- aac(3)-IIa- tetB*	2 (4.9%)
VII	*bla*_*NDM*_*- bla*_*OXA−*_ *bla*_*TEM−*_ *bla*_*IMP−*_ *mcr-2- aac(3)-IIa- tetB*	2(4.9) %
VIII	*bla*_*NDM*_*- bla*_*OXA−*_ *bla*_*TEM−*_ *tetB*	2(4.9) %
IX	*bla*_*NDM*_*- bla*_*OXA−*_ *bla*_*TEM−*_ *tetB*	1(2.4) %
X	*bla*_*NDM*_*- bla*_*OXA−*_ *bla*_*TEM*_*- mcr-2- aac(3)-Ia- tetB*	1(2.4) %
XI	*bla*_*NDM*_*- bla*_*OXA−*_ *bla*_*TEM−*_ *bla*_*VIM−*_ *mcr-2- tetB*	1(2.4) %
XII	*bla*_*OXA−*_ *bla*_*TEM−*_ *- mcr-1- mcr-2- aac(3)-IIa- tetB*	1 (2.4) %
XIII	*bla*_*OXA−*_ *bla*_*TEM−*_ *- bla*_*VIM−*_ *mcr-1- mcr-2- aac(3)-IIa- tetB*	1 (2.4) %
XIV	*bla*_*NDM*_*- bla*_*TEM−*_ *- bla*_*VIM−*_ *bla*_*KPC−*_ *mcr-1- mcr-2- aac(3)-IIa- tetB*	1 (2.4) %
XV	*bla*_*NDM*_*- bla*_*TEM−*_ *- bla*_*VIM*_ *- mcr-1- mcr-2- aac(3)-IIa- tetB*	1 (2.4) %
XVI	*bla*_*NDM*_*- bla*_*OXA−*_ *bla*_*VIM*_ *- mcr-2- aac(3)-IIa- tetB*	1(2.4) %
XVII	*bla*_*NDM*_*- bla*_*OXA−*_ *mcr-1- aac(3)-IIa- tetB*	1(2.4) %
XVIII	*bla*_*NDM*_*- bla*_*OXA−*_ *bla*_*TEM−*_ *- bla*_*VIM*_ *- mcr-2- aac(3)-Ia - aac(3)-IIa- tetB*	1 (2.4) %
VIII	*bla*_*NDM*_*- bla*_*OXA−*_ *bla*_*TEM−*_ *- bla*_*VIM*_ *- bla*_*IMP−*_ *mcr-2- aac(3)-IIa- tetB*	1(2.4) %
XIX	*bla*_*NDM*_*- bla*_*OXA−*_ *bla*_*TEM−*_ *- bla*_*VIM*_ *- aac(3)-Ia - aac(3)-IIa- tetB*	1(2.4) %
XX	*bla*_*NDM*_*- bla*_*OXA−*_ *bla*_*TEM−*_ *- bla*_*VIM*_ *- aac(3)-IIa- tetB*	1(2.4) %
XXI	*bla*_*NDM*_*- bla*_*OXA−*_ *bla*_*TEM−*_ *- bla*_*VIM*_ *-bla*_*IMP−*_ *bla*_*KPC−*_ *mcr-1- mcr-2- aac(3)-IIa-tetB*	1(2.4) %
XXII	*bla*_*NDM*_*- bla*_*OXA−*_ *bla*_*TEM−*_ *- bla*_*IMP−*_ *mcr-1- mcr-2- aac(3)-Ia- aac(3)-IIa- tetB*	1(2.4) %
XXIII	*bla*_*NDM*_*- bla*_*OXA−*_ *bla*_*TEM−*_ *- bla*_*VIM*_ *- bla*_*KPC*_ *mcr-1- mcr-2- aac(3)-Ia- aac(3)-IIa- tetB*	1(2.4) %
XXIV	*bla*_*NDM*_*- bla*_*OXA−*_ *bla*_*TEM−*_ *- bla*_*KPC−*_ *mcr-1- aac(3)-IIa- tetB*	1(2.4) %
XXV	No resistance genes	0%

The distribution of AMR-encoding genes with corresponding AMR phenotypes among *K pneumoniae* isolates is demonstrated in Table 5. The matching revealed similar phenotypic susceptibility patterns of amoxicillin-clavulanate, ceftriaxone, cefoxitin, cefuroxime, ceftazidime, piperacillin tazobactam, imipenem, meropenem, gentamycin, and amikacin the isolates versus coexisting resistance encoding genes (*bla*_*NDM*_, *bla*_*OXA*,_ *bla*_*IMP*,_ *bla*_*KPC*_, *bla*_*VIM*_, *and aac(3)-Ia*) and resistance pattern. while there was a different distribution pattern for *bla*_*TEM*,_ and resistance pattern to amoxicillin–clavulanate, ceftriaxone, cefoxitin, cefuroxime, ceftazidime, piperacillin tazobactam, imipenem, meropenem. There was a different distribution pattern for *mcr-1* and *mcr-2* resistance encoding genes and resistance pattern to colistin and for *aac(3)-IIa* and resistance pattern to gentamycin and amikacin.

**Table 5:** AMR testing of *K. pneumoniae* isolates with corresponding resistance Genes.

Prevalence of target genes among K. pneumoniae isolates NO. (%)		Antimicrobial Susceptibility Testing of resistant K. pneumoniae Isolates using Kirby–Bauer disc diffusion method							
		Amoxicillin -clavulanate NO. (%)	Ceftriaxone NO. (%)	Cefoxitin NO. (%)	Cefuroxime NO. (%)	Ceftazidime NO. (%)	Piperacillin-tazobactam NO. (%)	Imipenem NO. (%)	Meropenem NO. (%)
**ESBLs resistance encoding genes**									
*bla*_*TEM*_ 38(92.7)		37 (97.4)	35 (92.1)	33(86.8)	37(97.4)	33 (86.8)	32 (84.2)	26 (68.4)	30 (78.9)
**Carbapenem resistance encoding genes**									
*bla*_*NDM*_	39(95.1)	38(97.4)	36(92.3)	34(87.2)	38(97.4)	34(87.2)	33(84.6)	27 (69.2)	30 (76.9)
*bla*_*OXA*_	39(95.1)	38 (97.4)	36 (92.3)	34 (87.2)	38(97.4)	34 (87.2)	33 (84.6)	27 (69.2)	30 (76.9)
*bla*_*VIM*_	16(39)	16(100)	15(93.8)	15(93.8)	16(100)	15(93.8)	15(93.8)	15 (93.8)	15 (93.8)
*bla*_*IMP*_	8(19.5)	8 (100)	8 (100)	8 (100)	8 (100)	8 (100)	8 (100)	6 (75)	7 (87.5)
*bla*_*KPC*_	6(14.6)	6 (100)	5 (83.3)	5 (83.3)	6 (100)	5 (83.3)	5(83.3)	5 (83.3)	5 (83.3)
**Colistin resistance genes**		**Colistin**							
*mcr-1*	29(70.7)	24(82.8)							
*mcr-2*	27(65.9)	18(66.7)							
**Aminoglycoside resistance genes**		**Gentamycin**			**Amikacin**				
*aac(3)-Ia*	5(12.2)	5(100)			5(100)				
*aac(3)-IIa*	35 (87.8)	26(74.3)			24(68.6)				

### Genotypic profiles of CRISPER-CAS system encoding genes among *K. pneumoniae* isolates

PCR for detecting *CRISPR1*, *CRISPR2*, *CRISPR3*,* Cas1* and *Cas3*, revealed that 21(51.2%) harbored the *CRISPR1* gene, 19(46.3) harbored the *CRISPR2* gene, 26(63.4%) harbored the *CRISPR3* gene, 28(68.3%) of isolates harbored the *Cas1* gene and 23(56.1%) harbored the *Cas3* gene (Table 6).

**Table 6:** Molecular detection of CRISPER/CAS system encoding genes among *K. pneumoniae* isolates.

CRISPR encoding genes	No. (%)
*CRISPR3*	26 (63.4)
*CRISPR1*	21 (51.2)
*CRISPR2*	19 (46.3)
*Cas1*	28 (68.3)
*Cas3*	23 (56.1)

The presence or absence of CRISPR-Cas systems was shown where cas genes (cas1 or cas3) are present in conjunction with one of CRISPR arrays (CRISPR1, CRISPR2, CRISPR3). Out of 41 *K. pneumoniae* isolates, 27 (65.9%) were CRISPR-Cas-positive while 14 (34.1%) were.

**Fig. 3:**
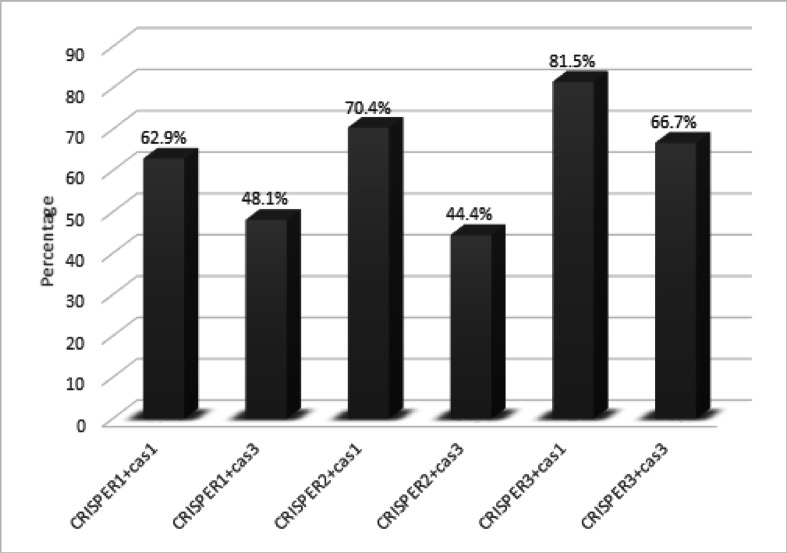
The distribution of different genotypes of CRISPR-Cas system.

CRISPR-Cas-negative. The distribution of different genotypes of CRISPR-Cas system is shown in Fig. 3.

Genotypic profiles of CRISPR-Cas system encoding genes among *K. pneumoniae* isolates are presented in Table 7 and revealed that 21.9% of the isolates coharbored *CRISPR1*, *CRISPR2*,* CRISPR3*,* Cas1*, and *Cas3*.

**Table 7:** Genotypic profiles of CRISPR-Cas system encoding genes among *K. pneumoniae* isolates.

Profile	CRISPR1	CRISPR2	CRISPR3	Cas1	Cas3	No. of isolates (%)	Presence of CRISPR-Cas system
**1**	**+**	**+**	**+**	**+**	**+**	**9(21.9%)**	**Present**
**2**	**+**	**-**	**-**	**-**	**-**	**4(9.8%)**	**Absent**
**3**	**-**	**+**	**+**	**+**	**-**	**4(9.8%)**	**Present**
**4**	**-**	**-**	**-**	**+**	**-**	**3(7.3%)**	**Absent**
**5**	**-**	**-**	**-**	**-**	**+**	**3(7.3%)**	**Absent**
**6**	**-**	**-**	**+**	**-**	**+**	**3(7.3%)**	**Present**
**7**	**+**	**-**	**+**	**+**	**+**	**3(7.3%)**	**Present**
**8**	**-**	**+**	**+**	**+**	**+**	**3(7.3%)**	**Present**
**9**	**-**	**-**	**-**	**-**	**-**	**2(4.9%)**	**Absent**
**10**	**+**	**+**	**+**	**+**	**-**	**2(4.9%)**	**Present**
**11**	**-**	**-**	**+**	**-**	**-**	**1(4.1%)**	**Absent**
**12**	**-**	**-**	**-**	**+**	**+**	**1(4.1%)**	**Absent**
**13**	**+**	**-**	**-**	**+**	**+**	**1(4.1%)**	**Present**
**14**	**+**	**-**	**+**	**+**	**-**	**1(4.1%)**	**Present**
**15**	**+**	**+**	**-**	**+**	**-**	**1(4.1%)**	**Present**

### Association between AMR detected by phenotypic Kirby–Bauer disc diffusion method and prevalence of the CRISPR-Cas system encoding genes

The AMR detected by Kirby–Bauer disc diffusion method among *K. pneumoniae* isolates was clearly higher in CRISPR-Cas-positive isolates compared to CRISPR-Cas-negative isolates. There was a non-significant positive correlation between the presence of CRISPR-Cas system and the existence of resistance in *K. pneumoniae* by Kirby–Bauer disc diffusion method (Pearson Chi-Square test, *p*- values = 0.1) (Table 8).

**Table 8:** Association of CRISPR-Cas system with the resistance in *K. pneumoniae*.

Gene	Distribution of Resistance Rate (%)		*P* value
	CRISPR-Cas positive (*N* = 27) No (%)	CRISPR-Cas negative (*N* = 14) No (%)	
Resistant isolates	25(92.6)	11(78.6)	**0.1**
Sensitive isolates	2 (7.4)	3 (21.4)	

Pearson’s Chi-square test was used to examine the association of CRISPR-Cas system with the AMR in *K. pneumoniae*. *A *p*-value < 0.05 was considered significant.

**Fig. 4:**
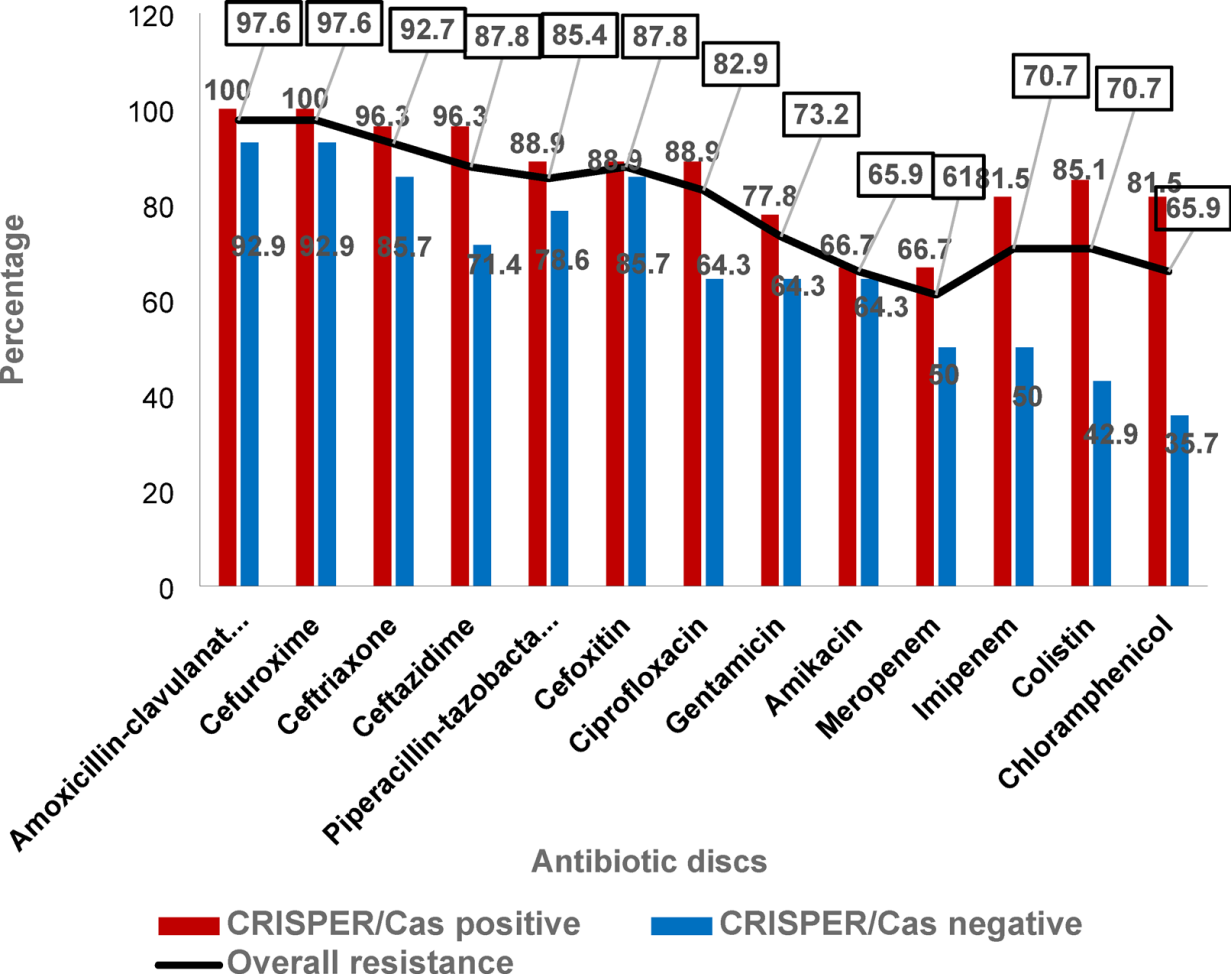
The prevalence of AMR detected by Kirby–Bauer disc diffusion method, among CRISPR-Cas-positive and -negative *K. pneumoniae* isolates.

CRISPR-Cas-positive isolates exhibited resistance to a broader range of antimicrobial agents, including amoxicillin-clavulanate (100%), cefuroxime (100%), ceftriaxone (96.3%), ceftazidime (96.3%), piperacillin-tazobactam (88.9%), cefoxitin (88.9%), ciprofloxacin (88.9%), gentamicin (77.8%), amikacin (66.7%), meropenem (66.7%), imipenem (81.5%), colistin (85.1%), and chloramphenicol (81.5%). There was a significant difference of AMR of imipenem, colistin and chloramphenicol (Pearson Chi-Square test, *p*-values = 0.047, 0.005, and 0.012, respectively) among CRISPR-Cas-positive and CRISPR-Cas - negative *K. pneumoniae* isolates as shown in Fig. 4.

### Association between AMR encoding genes detected by PCR and prevalence of the CRISPR-Cas system encoding genes

The percentage of *K. pneumoniae* isolates harboring AMR encoding genes was clearly higher in the CRISPR-Cas-positive isolates than in the CRISPR-Cas-negative isolates except for *bla*_NDM_, *bla*_OXA_ and *bla*_IMP_ encoding genes. The percentage of presence of *bla*_NDM,,_ *bla*_OXA_ and *bla*_IMP_ in CRISPR-Cas-positive isolates was 92.6%, 92.6%, and 14.8%, respectively while it was 100%, 100% and 28.6%, respectively in CRISPR-Cas-negative isolates.

For other AMR encoding genes CRISPR-Cas-positive *K. pneumoniae* isolates exhibited higher percentage of resistant genes compared to CRISPR-Cas-negative isolates. The presence of *bla*_TEM_, *aac(3)-IIa*,* mcr-1*,* mcr-2*, *bla*_VIM_, *bla*_KPC_, *and aac(3)-Ia* genes was observed in 100%, 96.3%, 81.5%, 66.7%, 44.4%, 22.2%, and 14.8% of isolates, respectively.

**Fig. 5:**
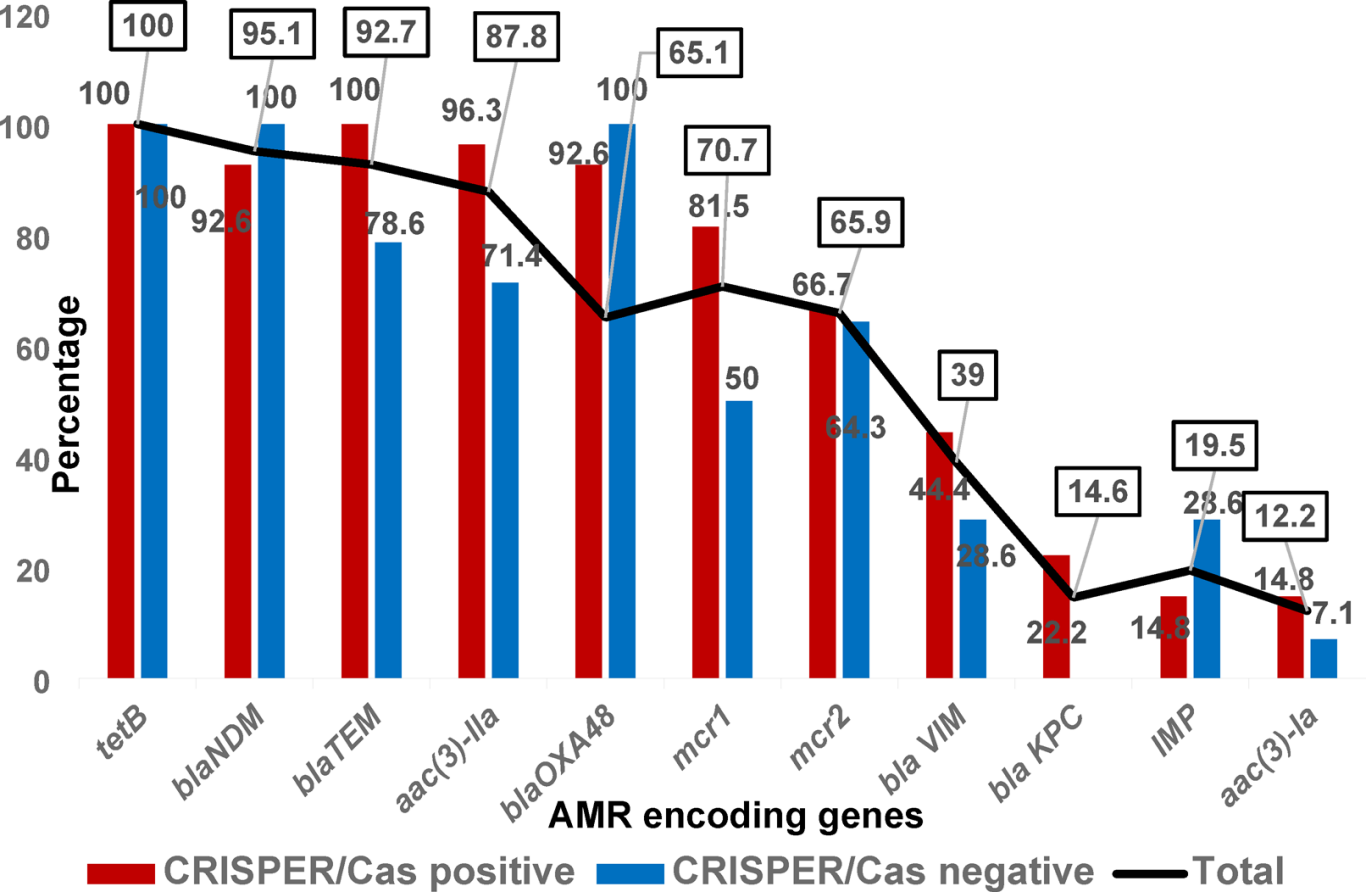
The prevalence of AMR encoding genes among the CRISPR-Cas-positive and CRISPR-Cas-negative *K. pneumoniae* isolates.

There was a significant difference (*p*-value < 0.05) of AMR encoding genes; *aac(3)-IIa* and *mcr-1* (Pearson Chi-Square test, *p*-values = 0.039 and 0.036, respectively) among CRISPR-Cas-positive and CRISPR-Cas - negative *K. pneumoniae* isolates which were found in 96.3% and 81.5% of the CRISPR-Cas positive isolates, respectively versus 71.4% and 50% of the CRISPR-Cas negative isolate as shown in Fig. 5.

**Fig. 6:**
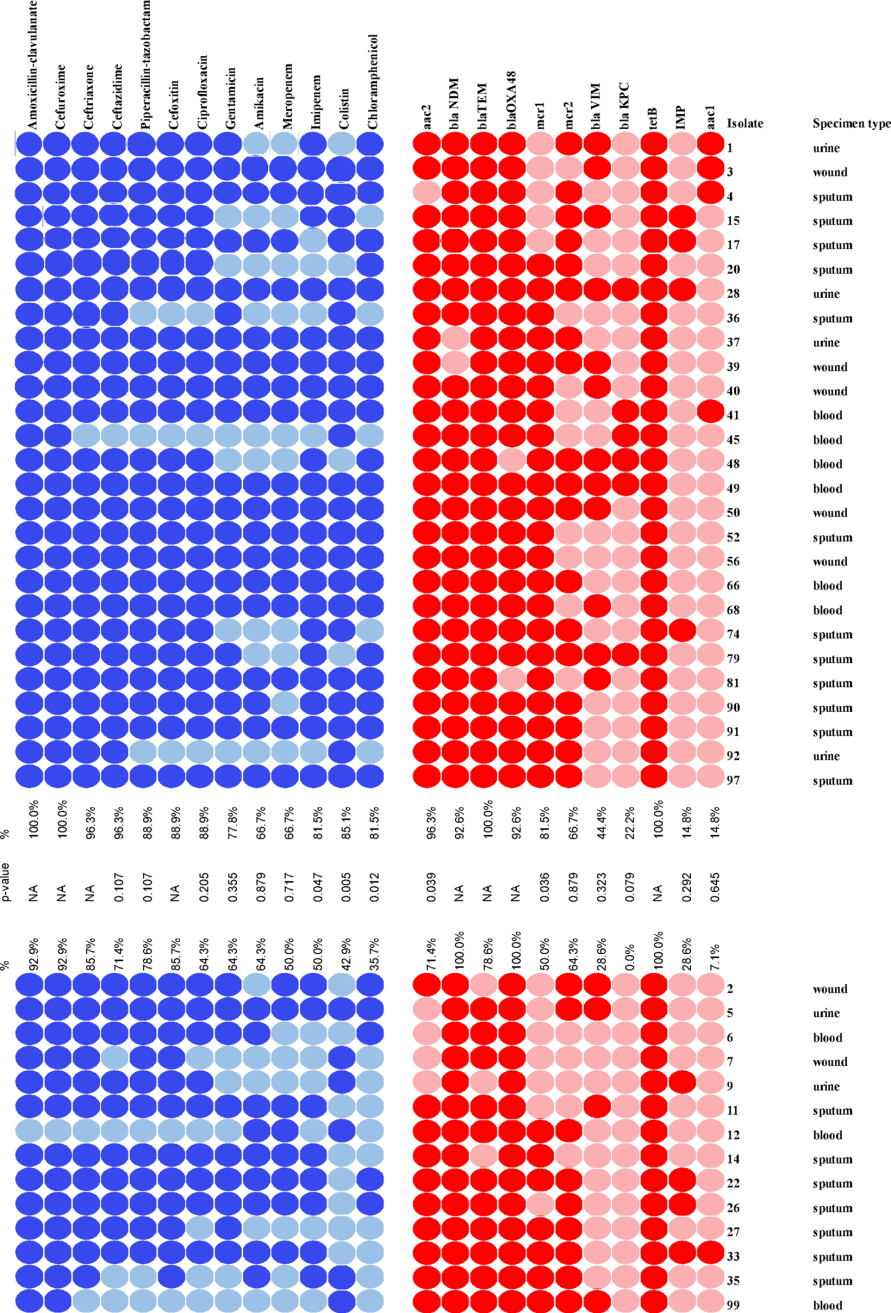
A heatmap displaying the AMR profiles and genetic determinants among the isolates carrying CRISPR-Cas (Upper panel) versus CRISPR-Cas negative (Lower panel) *Red and pink colors indicate the presence and absence of the ARGs*,* respectively; Dark and light blue colors indicate phenotypic resistance and susceptibility to the tested antimicrobial agents*,* respectively.*

Phenotypic resistance and susceptibility to the tested antimicrobial agents, together with the presence or absence of the ARGs and their correlation with the presence of CRISPR-Cas system are displayed in a heatmap **(**Fig. 6**)**; the isolates carrying CRISPR-Cas are in upper panel while CRISPR-Cas negative isolates are in lower panel.

## Discussion

*K. pneumoniae* is among the top pathogens causing nosocomial infections worldwide^32^. *K. pneumoniae’s* pan-genome is around five to six Mbp in size^15^, The size and fluidity of the accessory genome are key indicators of a species’ adaptability. Species with a large, “open” pan-genome (like *E. coli* or *Klebsiella pneumoniae*) are highly versatile and can rapidly acquire new traits, which is why they can be significant threats in clinical and environmental settings. In contrast, species with a smaller, “closed” pan-genome tend to be more specialized and have less genetic diversity^33^.

*K. pneumoniae* has become increasingly resistant to antibiotics, making antibiotic therapy more challenging, several antimicrobial resistance genes were discovered in *K. pneumoniae* for the first time before spreading to other pathogens e.g. *bla*_KPC_, *bla*_OXA−48,_ and *bla*_NDM−_^34^.

The CRISPR-Cas systems are involved in limiting the entry of foreign DNA to bacteria and archaea and have also been related to the expression of virulence factors in bacteria^32^. In the current study, we aimed to detect the prevalence of CRISPR-Cas encoding genes in MDR clinical isolates of *K. pneumoniae* and analyze the correlation between the presence of CRISPR-Cas system and prevalence of the AMR detected by phenotypic and molecular techniques as limited researches are being carried out in the Middle East to address this correlation.

In the current study, antimicrobial susceptibility testing by Kirby–Bauer disc diffusion methods revealed that 95% of *K. pneumoniae* isolates were MDR. The results of this study were in accordance with two studies who found that 90.2% and 94% of *K. pneumoniae* isolates were MDR^35,36^. Previous studies conducted in Egypt found that the rate of MDR Gram-negative isolates was 85.6%, 66.5% and 93.6%^37–39^. Moreover, a recent study conducted in Egypt revealed that the rate of MDR Gram-negative isolates was 98.2% that was higher than the results of this study^40^.

*K. pneumoniae* isolates in the current study showed high AMR against amoxicillin-clavulanate (98%) and piperacillin-tazobactam (91%), 2nd and 3rd generation cephalosporins, cefoxitin (90%), cefuroxime (98%), ceftriaxone (97%), and ceftazidime (94%). High resistance rates to these antimicrobials were also observed in other studies^41,42^.

Also, considerable carbapenem resistance was detected in the current study, the resistance rate to meropenem and imipenem was 78% and 75%, respectively. This is higher than a previous study conducted in Egypt, which reported a resistance rate of 50% to meropenem^43^. Another study in Egypt showed that the resistance rate was 31.3% and 30% to imipenem and meropenem, which was lower than this study^44^. Likewise, Baral et al. (2024) from Nepal showed that 61.1% and 69.4% of *K. pneumoniae* isolates were resistant to meropenem and imipenem respectively, which is comparable to our results^45^.

Chloramphenicol is no longer the drug of choice for many infections. Its usage in the developed world is limited to life-threatening infections without safer alternatives. However, it is still commonly used in many parts of the developing world^46^.

In the current study, *K. pneumoniae* isolates showed moderate incidence of resistance to chloramphenicol (46%), this is consistent with the findings of a study from Egypt reported that the frequency of resistance to chloramphenicol was 54%^36^.

Microbroth dilution results of meropenem, amikacin reveled that the percentage of meropenem resistance was 67% which was lower than that found in other studies reported 100% resistance to meropenem by MIC^47,48^. The resistance rate to amikacin by MIC was 76%, this result was comparable to a study showed a resistance of 50% to amikacin^49^. The moderate resistance to amikacin may be due to its less frequent use as empirical therapy and the absence of considerable cross-resistance with β-lactam antimicrobials^50^.

Although colistin is now used as a “last resort” for treating infections caused by some of the most virulent MDR Gram-negative bacteria^51^. Determination of MIC provide more accurate results for colistin susceptibility testing compared to disc diffusion methods because colistin is a large molecule that does not diffuse well in agar, leading to inconsistent results^52^. The *K. pneumoniae* isolates in the current investigation had a significant incidence of colistin resistance (41%) by determination of MIC. Several studies conducted in Egypt reported lower rates of colistin resistance that were 4.9%, 7.5 and 17.2%^53–55^. This resistance rate could be because of colistin therapy that was discontinued many years ago, is now used as a last-resort antibiotic for carbapenem resistant *K. pneumoniae* infections.

The high MDR rate could be a result of misuse and abuse of antibiotics in Egypt. The increasing rate of these isolates emphasizes the importance of choosing an appropriate antimicrobial regimen based on antibiotic susceptibility testing^56^. Numerous causes, including the use of antibiotics in healthcare facilities, the community, farm animals, agriculture, and the environment, all contribute to the rise of antibiotic resistance. Antibiotics are overused because they may be purchased easily over the counter without a prescription^57^. Thus, antibiotic usage must be monitored closely to reduce antibiotic misuse, as antibiotic resistance is spreading quickly^58^.

In the current study, forty-one isolates were selected for molecular investigation where the prevalence of ESBL encoding gene; *bla*_TEM_ was 92.7%. Other studies revealed that the frequency of *bla*_TEM_ was 100% and 52%^59,60^. ESBL-producing *Enterobacteriaceae* carry a broad-spectrum beta-lactamase enzyme that enables them to become resistant to nearly all antibiotics in the penicillin and cephalosporin classes. In such cases, the remaining treatment option is an antibiotic from the carbapenem family, which contributes to the development of carbapenem resistance^61^.

The current study reported higher frequencies of carbapenem resistance encoding genes among *K. pneumoniae* isolates, including *bla*_NDM_ and *bla*_OXA−48_ genes (95.1%). These results were significantly higher than several studies including a study from Egypt reported that the incidence of *bla*_NDM_ and *bla*_OXA−48_ was 70% and 52%, respectively^62^. Another study found that 66.67% and 62.50% of carbapenem resistant *Enterobacteriaceae* isolates possessed *bla*_OXA*−48*_ and *bla*_NDM_, respectively^63^. In contrast, the prevalence of *bla*_VIM,_ *bla*_IMP_, and *bla*_KPC_ in this study was 39%, 19.5%, and 14.6%, respectively. Similar to our results a study in Egypt showed that the prevalence of *bla*_VIM_ and *bla*_IMP_ were 11.1% and 0%, respectively^64^. Another study in Egypt, revealed that *bla*_OXA*−48*_ was the prevalent gene (15.5%), followed by *bla*_VIM_ (15%), *bla*_IMP_ (7.5%), *bla*_KPC_ (4%), and *bla*_NDM_ (3.8%).

The rationale behind the expression-discrepancy of carbapenem resistance genes with crossponding phenotypes is not well-understood^65^. It is possible that the antibiotic itself may modulate the ARGs into low in vitro expression^66^, or that the heteroresistance phenomena associated with unstable tandem gene amplification, or rare mutation such as frameshift mutations associated with upregulation of the MexXY-OprM pump, loss of OprD expression^67^ and environmental modulation to the resistant genes, all these factors may explain the exhibited phenotypic carbapenem susceptibilities^65^.

Untreatable infections due to carbapenem resistant *K. pneumoniae* are on the rise among patients in medical facilities^68^. With the increasing use of carbapenems in clinical practice, the emergence of carbapenem-resistant pathogens now poses a great threat to human health. Currently, antibiotic options for the treatment of carbapenem resistant *K. pneumoniae* are very limited^69^.

In this study, *aac(3)-IIa* was the most prevalent aminoglycoside resistance encoding gene at 87.8%, followed by aac*(3)-Ia* at 12.2%. The combination of both genes was observed in four isolates. One study reported a lower frequency for the two *aac (3)-II* genes that was 79.3% and 64%, respectively^70^. In contrast, Kadkhoda et al. (2024) found that the percentage of *aac(3)-Ia* was 29.5% while *aac(3)-IIa* genes weren’t detected^71^.

The *mcr-1* gene is the most commonly to cause colistin resistance in humans, also *mcr-2*, has a role in the development of colistin resistance^72^.

The results of the current study revealed that 70.7% of the *K. pneumoniae* isolates were harboring *mcr-1* gene while 65.9% were harboring *mcr-2* gene. This high prevalence rate of *mcr-1* and *mcr-2* genes was in accordance with the high resistance detected by the phenotypic determination of MIC (41%). One study from Egypt reported that 84.4% of their *K. pneumoniae* isolates harbored *mcr-1* encoding gene^72^. On the other hand, many studies revealed lower rates of *mcr-1* which were 4%, 4.2% and 5%, respectively and no one of their isolates had *mcr-2*^53,73,74^. The *mcr-1* and *mcr-2* genes dissemination makes it necessary to search for alternatives to substitute ineffective antibiotics^72^.

Due to dissemination of tetracycline resistance in our region^75,76^ the tetracycline resistance gene (*tet B*) was included in our study as a key molecular marker to investigate the mobility of resistance determinants and their relationship with the CRISPR-Cas system that is separate from the phenotypic antimicrobial susceptibility testing as it is not a first-line or routinely used therapeutic option for treating infections caused by MDR *K. pneumoniae* isolates.

The *tetB* gene generally was reported as the most common gene among tetracycline-resistant *Enterobacteriaceae*^77^. In the present study, *tetB* gene was detected in 100% of isolates. Other studies detected *tetB* gene in 42% and 18.4% in their *K. pneumoniae* isolates^78,79^.

These findings show a remarkable frequency of *tetB* gene distribution and subsequent tetracycline resistance. This may be due to long-term and widespread use of tetracycline in animal farms and agriculture for growth promotion purposes. Also, the *tet* genes are located on highly mobile genetic elements as plasmids, transposons, and conjugative transposons which are responsible for the horizontal transfer of ARGs, contributing to the widespread distribution of tetracycline resistance in *K. pneumoniae*^80^.

Pearson’s Chi-square test or Fisher’s exact test are both used to compare observed and expected frequencies in a contingency table, determining if differences are due to chance or not, with Pearson’s being an approximation suitable for larger sample sizes and Fisher’s providing an exact calculation for low number samples^81^.

The results of this study indicated a significant agreement between resistance profile identified by Kirby–Bauer disc diffusion method and the presence of resistance encoding genes identified by PCR in *K. pneumoniae*. We found that the phenotypic resistance of amoxicillin-clavulanate, ceftriaxone, cefoxitin, cefuroxime, ceftazidime, piperacillin-tazobactam, imipenem, and meropenem among *K. pneumoniae* isolates correlate with the presence of *bla*_NDM_, *bla*_OXA_, *bla*_TEM_, *bla*_VIM_, *bla*_IMP_, and *bla*_KPC_ genes. This correlation between phenotypes and genotypes were in agreement with recent studies^82,83^.

*K. pneumoniae* has developed antimicrobial resistance to many antibiotics due to high selection pressure from increasing use and misuse of antibiotics over the years. The transmission and acquisition of AMR occur primarily via a human-to-human interface within or outside of healthcare facilities^84^. In order to combat AMR, rational antibiotic prescription, limited use of prophylactic antimicrobials, patients’ education, compliance with antibiotic therapy, and appropriate hospital hygiene through effective antimicrobial stewardship are necessary^85^.

The CRISPR-Cas systems, derived from the adaptive immune system of prokaryotes, has been found in approximately 50% of bacterial genomes and 87% of archaea^1^. It is worth noting that pathogens with CRISPR-Cas systems were less likely to carry antibiotic resistance genes than those lacking this defense system^2^. However, recent studies have shown that the CRISPR systems are sometimes missed or inactivated and may not be an effective barrier to plasmid and drug resistance spread^86^. Therefore, the analysis of the relationship between the CRISPR-Cas system and antibiotic resistance will help to better understand the mechanism of bacterial resistance and provide new directions for the prevention and combating bacterial resistance^87^.

In this study, 65.9% (27/41) of *K. pneumoniae* isolates harbored CRISPR-Cas system encoding genes, which is considered a high percentage. This is higher than a previous report from Egypt that found CRISPR-Cas in 25.4% of their collection isolates^88^.

The high prevalence of the CRISPR-Cas systems in MDR *K. pneumoniae* in this study could be contrary to the capability of CRISPR-Cas to accurately eliminate drug resistance-related genes from bacterial strains in populations and to re-sensitize bacteria to antibiotics by deleting AMR encoding genes. The presence of CRISPR-Cas system encoding genes in resistant bacteria can indicate that these systems were present before but become inactive during evolution in order to give the bacteria the chance to host AMR genes and become resistant^89^.

In the current study, we found diverse profiles for CRISPR-Cas systems, the most prevalent one was *CRISPR1*,* CRISPR2*,* CRISPR3*,* cas1*,* and cas3* (21.9%). This was in agreement with Makarova et al., who revealed that the CRISPR arrays are highly variable among bacterial species^3^.

Two *cas* genes were detected in this study. These genes included cas1 (68.3%), a universal *cas* gene that is found in most CRISPR-Cas types, and cas3(56.1%), the signature gene of the type I CRISPR-Cas system^88,90^.

No typical significant positive correlation between AMR and the presence of CRISPR-Cas system was detected (*P* value 0.1). The percentage of resistant *K. pneumoniae* isolates was clearly higher in the CRISPR-Cas-positive isolates than in the CRISPR-Cas-negative isolates.

The CRISPR system may be present as a remnant of a previous encounter with phages or foreign DNA, but it is inactive. Additionally, the CRISPR system requires the presence of specific triggering elements, such as protospacer adjacent motifs and foreign nucleic acids, to become active. Absence of these elements may render the CRISPR system inactive^91,92^. Additionally, mutations in genes encoding CRISPR-associated proteins or in the regulatory sequences of the CRISPR array could render the system inactive. Moreover, environmental conditions may play a role in regulating the activity of the CRISPR system. For example, nutrient availability or stress conditions could influence the activation of the system^93,94^.

Similar to our results Alkompoz et al. found a weak, non-significant positive correlation between the number of CRISPR system spacers and the number of resistance plasmids and ARGs in *K. pneumoniae* isolates^88^. Also two studies conducted in China found a negative correlation between the acquisition of ARGs and the presence of CRISPR-Cas systems^87,95^.

In the current study, the AMR detected by Kirby–Bauer disc diffusion method among *K. pneumoniae* isolates was higher in CRISPR-Cas-positive isolates compared to CRISPR-Cas-negative isolates indicating a potential association between the presence of the CRISPR-Cas system and increased antimicrobial resistance in *K. pneumoniae*.

A study from Egypt found that 25.4% of *K. pneumoniae* isolates carried the CRISPR-Cas system, and these isolates were more likely to be MDR or XDR compared to CRISPR-Cas-negative isolates. However, there was non-significant difference in susceptibility to the tested antimicrobial agents among the two groups of isolates^88^.

On the other hand, another study reported that resistance to various antibiotic classes, including β-lactams, quinolones, aminoglycosides, tetracyclines, and β-lactam/enzyme inhibitor combinations, was higher in the absence of the CRISPR-Cas system among *K. pneumoniae* isolates^95^. Similarly, a study in Iraq demonstrated an inverse association between the presence of the CRISPR-Cas system and antibiotic resistance, including ESBL production and carbapenem resistance in *K. pneumoniae* isolates and reported that multidrug resistance, extensive drug resistance, and pandrug resistance were more prevalent in CRISPR-Cas-negative strains^29^.

In the current study, the percentage of *K. pneumoniae* isolates harbored AMR encoding genes was clearly higher in the CRISPR-Cas-positive isolates than in the CRISPR-Cas-negative isolates except for *bla*_NDM_, *bla*_OXA_ and *bla*_IMP_ encoding genes. There was a significant difference (*p*-value < 0.05) of AMR encoding genes; *aac(3)-IIa* and *mcr-1* (Pearson Chi-Square test, *p*-values = 0.039 and 0.036, respectively) among CRISPR-Cas-positive and CRISPR-Cas - negative *K. pneumoniae* isolates which were found in 96.3% and 81.5% of the CRISPR-Cas positive isolates, respectively versus 71.4% and 50% of the CRISPR-Cas negative isolate.

Generally, the CRISPR-Cas systems found in *K. pneumoniae* are not always correlated to the absence of ARGs; rather, an enormous number of ARGs and CRISPR-Cas systems have been found co-existing in the analyzed genomes^96^.

Alkompoz et al. revealed that the frequency of the genes, including *bla*_VIM_, *bla*_NDM_, and *tetB* were significantly higher in the presence of CRISPR-Cas systems. However, other genes such as *bla*_TEM_ and *bla*_KPC_ were significantly higher in the genomes of the CRISPR-Cas negative strains^88^. Also, Kadkhoda et al. found that the frequency of the *bla*_TEM_ was significantly lower in the isolates with CRISPR-Cas- positive isolates in comparison to CRISPR-Cas- negative isolates. There was a significant inverse correlation between the presence of ESBL and some AME genes with CRISPR-Cas-positive isolates^71^.

The impact of the CRISPR-Cas system on limiting the dissemination of ARGs and, consequently, antimicrobial resistance was evident from some studies conducted on other bacterial species. The CRISPR-Cas system was previously found to be significantly associated with the absence of ARGs and high drug susceptibility in *Enterococcus faecalis*^97,98^and *Pseudomonas aeruginosa*^99^. On the other hand, several studies demonstrated that there was a highly significant inverse association between the prevalence of CRISPR-Cas system and drug resistance in carbapenem-resistant and ESBL-producing *K. pneumoniae*^29,95,100^. There are a lot of reasons explaining why the existence of the CRISPR-Cas systems on the bacterial genome does not always impede the dissemination of ARGs, starting from the adaptation stage. Point mutations and insertion sequence-mediated mutations in the adaptation genes correlated with the spread of MDR strains, as reported before in *Shigella* species^101^. In addition, strong selective pressure for antibiotic resistance may result in CRISPR repression and many CRISPR-harboring strains may be immunologically inactive owing to the existence of self-targeting spacers, which would be expected to induce an autoimmune response and host cell death^102^. On the other hand, phages expressing anti-CRISPR proteins (Acrs) may inactivate the CRISPR-Cas system, resulting in the dissemination of ARGs, as found in *P. aeruginosa*^103^.

## Conclusion

The study revealed the high prevalence of MDR *K. pneumoniae* isolates in Egypt by phenotypic and molecular techniques. The study showed high resistance rates of *K. pneumoniae* to broad-spectrum β-lactam antibiotics, including third and fourth generation cephalosporins. Additionally, high resistance rates were noted for ciprofloxacin, gentamicin, amikacin, meropenem, and imipenem. In contrast, lower resistance rates were found for colistin and chloramphenicol. High prevalence of resistance encoding genes were detected, with a majority of isolates carrying the ESBLs encoding gene, carbapenem resistance encoding genes, *bla*_NDM_ and *bla*_OXA_, aminoglycosides resistance encoding gene; *aac(3)-IIa* genes, *tetB* and the colistin resistance encoding genes *mcr-1*. There was no overall significant positive correlation between resistance detected byphenotypic and genotypic methods and the presence of CRISPR-Cas system except for imipenem, colistin and chloramphenicol phenotypic resistance andaac(3)-IIa and mcr-1 genes however, the percentage of resistant K. pneumoniae isolates was clearly higher in the CRISPR-Cas-positive isolates than in theCRISPR-Cas-negative isolates.

The results of this study challenge the belief that CRISPR-Cas systems are effective in eliminating drug resistance genes and sensitizing bacteria to antibiotics by deleting antimicrobial resistance encoding elements. Analysis of the relationship between the CRISPR-Cas system and AMR will help to better understand the mechanism of bacterial resistance and provide new strategies for the prevention and treatment of bacterial AMR. However, our investigation was limited by the small number of non-MDR isolates, as most *Klebsiella pneumoniae* isolates were MDR. Future studies with a larger range of isolates, including non-MDR strains, will be required to thoroughly confirm the association.

## Supplementary Information

Below is the link to the electronic supplementary material.

Supplementary Material 1

## Data Availability

All generated or analysed data during this study are included in this published article and supplementary materials.

## Abbreviations

AMR: Antimicrobial resistance
Cas: CRISPR-associated genes/proteins
CRISPR: The clustered regularly interspaced short palindromic repeats
*K. pneumoniae*: *Klebsiella pneumoniae*
MDR: multidrug-resistant
PCR: Polymerase chain reaction
