# Supplementary material for: Association of the existence of CRISPR-Cas system and antimicrobial resistance in multi-drug resistant Klebsiella pneumoniae in Egypt
Source: Sci Rep. 2025 Nov 25;15:41814. doi: 10.1038/s41598-025-26706-6 (PMC12647879; doi:10.1038/s41598-025-26706-6)
Supplement: Supplementary file 1 — Supplementary Material 1 [file 41598_2025_26706_MOESM1_ESM.docx]

**Table S 1.** PCR thermal profile for detection of antimicrobial resistance encoding genes and CRISPER-CAS system encoding genes

| S Stage  Gene | Primary denaturation | Denaturation | Annealing | Extension | Terminal extension |
| --- | --- | --- | --- | --- | --- |
| *bla_KPC_* | 95°C- 5 min | 94°C- 30 sec | 51°C-30 sec | 72°C- 30 sec | 72°C- 7 min |
|  |  | 30 cycles | | |  |
| *bla_TEM_* | 95°C- 5 min | 94°C- 30 sec | 55°C-30 sec | 72°C- 1 min | 72°C- 7 min |
|  |  | 30 cycles | | |  |
| *bla_OXA_* | 95°C- 5 min | 94°C- 30 sec | 55°C-30 sec | 72°C- 1 min | 72°C- 7 min |
|  |  | 30 cycles | | |  |
| *bla_IMP_* | 95°C- 5 min | 95°C- 30 sec | 60°C- 30 sec | 72°C- 30 sec | 72°C- 7 min |
|  |  | 35 cycles | | |  |
| *bla_NDM_* | 95°C- 5 min | 95°C- 30 sec | 55°C-30 sec | 72°C- 50 sec | 72°C- 5 min |
|  |  | 30 cycles | | |  |
| *bla_VIM_* | 95°C- 2 min | 95°C- 15 sec | 52°C-20 sec | 72°C- 15 sec | 72°C-2 min |
|  |  | 30 cycles | | |  |
| *mcr-1* | 95°C- 5 min | 94°C- 25 sec | 52°C-25 sec | 72°C- 25 sec | 72°C- 7 min |
|  |  | 30 cycles | | |  |
| *mcr-2* | 95°C- 5 min | 94°C- 30 sec | 53°C-30 sec | 72°C- 30 sec | 72°C- 7 min |
|  |  | 30 cycles | | |  |
| *aac(3)-Ia* | 95°C- 5 min | 94°C- 30 sec | 59°C-30 sec | 72°C- 30 sec | 72°C- 7 min |
|  |  | 35 cycles | | |  |
| *aac(3)-IIa* | 95°C- 5 min | 95°C- 30 sec | 55°C-30 sec | 72°C- 50 sec | 72°C- 5 min |
|  |  | 30 cycles | | |  |
| *tetB* | 95°C- 5 min | 94°C- 25 sec | 52°C-25 sec | 72°C- 25 sec | 72°C- 7 min |
|  |  | 30 cycles | | |  |
| *CRISPR1* | 95°C- 5 min | 94°C- 30 sec | 55°C-30 sec | 72°C- 1 min | 72°C- 7 min |
|  |  | 30 cycles | | |  |
| *CRISPR2* | 95°C- 5 min | 94°C- 30 sec | 55°C-30 sec | 72°C- 1 min | 72°C- 7 min |
|  |  | 30 cycles | | |  |
| *CRISPR3* | 95°C- 5 min | 94°C- 30 sec | 55°C-30 sec | 72°C- 1 min | 72°C- 7 min |
|  |  | 30 cycles | | |  |
| *Cas1* | 95°C- 5 min | 94°C- 30 sec | 55°C-30 sec | 72°C- 1 min | 72°C- 7 min |
|  |  | 30 cycles | | |  |
| *Cas3* | 95°C- 5 min | 94°C- 30 sec | 55°C-30 sec | 72°C- 1 min | 72°C- 7 min |
|  |  | 30 cycles | | |  |

**Table S2.** Results of Kirby–Bauer disc diffusion susceptibility testing for *K. pneumoniae* clinical isolates

| **Antibiotics** | **Sensitive No. (%)** | **Intermediate No. (%)** | **Resistant**  **No. (%)** |
| --- | --- | --- | --- |
| Amoxicillin-clavulanate (20/10) µg | 1 (1%) | 1 (1%) | **98 (98%)** |
| Cefuroxime (30) µg | 1 (1%) | 1 (1%) | **98 (98%)** |
| Ceftriaxone (30) µg | 2 (2%) | 1 (1%) | **97 (97%)** |
| Ceftazidime (30) µg | 5 (5%) | 1 (1%) | 94 (94%) |
| Piperacillin-tazobactam (100/10) µg | 5 (5%) | 4 (4%) | 91 (91%) |
| Cefoxitin (30) µg | 6 (6%) | 4 (4%) | 90 (90%) |
| Ciprofloxacin (5) µg | 12 (12%) | 6 (6%) | 82 (82%) |
| Gentamicin (10) µg | 18 (18%) | 0 (0%) | 82 (82%) |
| Amikacin (30) µg | 17 (17%) | 4 (4%) | 79 (79%) |
| Meropenem (10) µg | 19 (19%) | 3 (3%) | 78 (78%) |
| Imipenem (10) µg | 19 (19%) | 6 (6%) | 75 (75%) |
| Colistin 10 µg | **33 (33%)** | 0 (%) | 67 (67%) |
| Chloramphenicol (30) µg | **48 (48%)** | 6 (6%) | 46 (46%) |

**Fig. S1. The distribution of K. pneumoniae isolates according to the type of specimen**

*
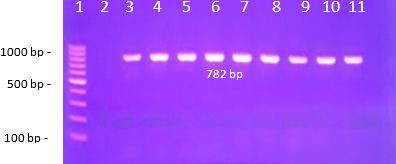
*

**Fig.S2. Agarose gel (1.5%) electrophoresis of amplified products of *bla*_NDM_ at ⁓ 782 bps. Lane 1: (1000bp) Marker, Lane 2: Negative control, Lane 3-11: Positive amplification of the gene.**

*
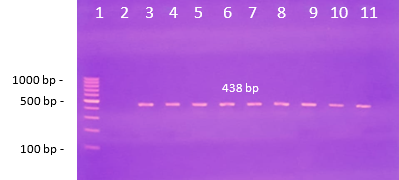
*

**Fig.S3 Agarose gel (1.5%) electrophoresis of amplified products of *bla*_OXA_ at ⁓ 438 bps. Lane 1: (1000bp) Marker, Lane 2: Negative control, Lane 3-11: Positive amplification of the gene.**

1 2 3 4 5 6 7 8 9 10 11

**
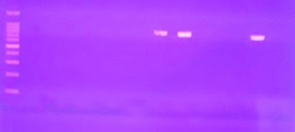
**

685 bp

**Fig.S4. Agarose gel (1.5%) electrophoresis of amplified products of *bla*_VIM_ at ⁓ 685 bps. Lane 1: (1500bp) Marker, Lane 2: Negative control, Lane 7, 8 and 11: Positive amplification of the gene.**

*
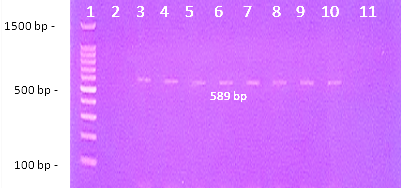
*

**Fig.S5. Agarose gel (1.5%) electrophoresis of amplified products of *bla*_IMP_ at ⁓ 589 bps. Lane 1: (1500bp) Marker, Lane 2: Negative control, Lane 3-10: Positive amplification of the gene.**

*
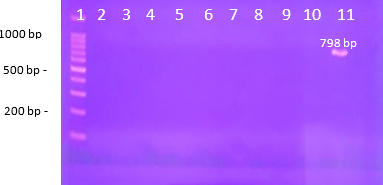
*

**Fig.S6. Agarose gel (1.5%) electrophoresis of amplified products of *bla*_KPC_ at ⁓ 798 bps. Lane 1: (1500bp) Marker, Lane 2: Negative control, Lane 11: Positive amplification of the gene.**

**
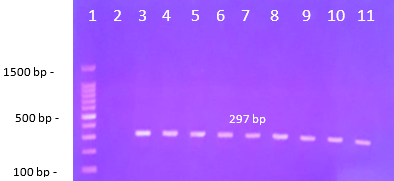
**

**Fig.S7. Agarose gel (1.5%) electrophoresis of amplified products of *bla*_TEM_ at ⁓ 297 bps. Lane 1: (1500bp) Marker, Lane 2: Negative control, Lane 3-11: Positive amplification of the gene.**


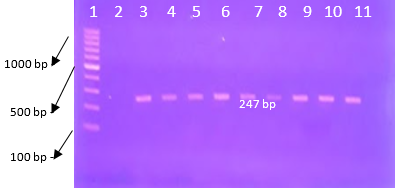


**Fig.S8. Agarose gel (1.5%) electrophoresis of amplified products of *aac(3)-IIa* at ⁓ 247 bps. Lane 1: (1000bp) Marker, Lane 2: Negative control, Lane 3-11: Positive amplification of the gene**.

1 2 3 4 5 6 7 8 9 10 11


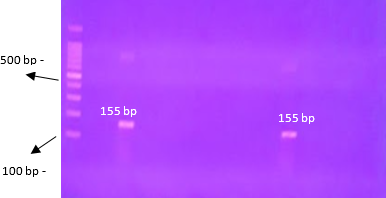


**Fig.S9. Agarose gel (1.5%) electrophoresis of amplified products of aac(3)-Ia**  **at ⁓ 155 bps. Lane 1: (1500bp) Marker, Lane 2: Negative control, Lane 3 and 9: Positive amplification of the gene.**

*
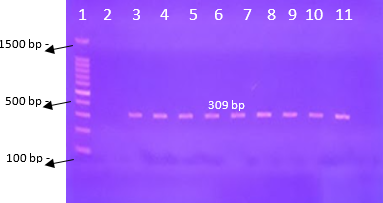
*

**Fig.S10. Agarose gel (1.5%) electrophoresis of amplified products of *mcr-1* at ⁓ 309 bps. Lane 1: (1500bp) Marker, Lane 2: Negative control, Lane 3-11: Positive amplification of the gene.**

*
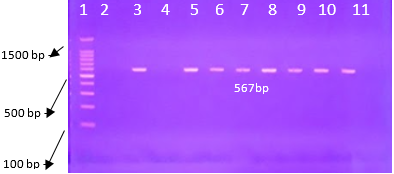
*

**Fig.S11. Agarose gel (1.5%) electrophoresis of amplified products of *mcr-2* at ⁓ 567 bps. Lane 1: (1500bp) Marker, Lane 2: Negative control, Lane 3, (5-11): Positive amplification of the gene.**

*
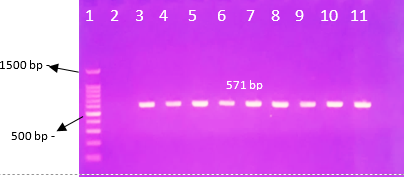
*

**Fig.S12. Agarose gel (1.5%) electrophoresis of amplified products of *tetB* at ⁓ 571 bps. Lane 1: (1500bp) Marker, Lane 2: Negative control, Lane 3-11: Positive amplification of the gene.**

1 2 3 4 5 6 7 8 9 10 11

1598 bp


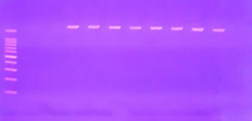


1 2 3 4 5 6 7 8 9 10 11

1598 bp

**Fig.S13. Agarose gel (1.5%) electrophoresis of amplified products of *CRISPR3* at ⁓ 1598 bps. Lane 1: (1500 bp) Marker, Lane 2: Negative control, Lane 4-11: Positive amplification of the gene.**

1 2 3 4 5 6 7 8 9 10 11


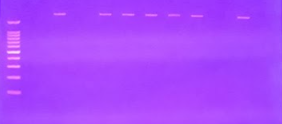


1888 bp

**Fig.S14. Agarose gel (1.5%) electrophoresis of amplified products of *CRISPR1* at ⁓ 1888 bps. Lane 1: (1500 bp) Marker, Lane 2: Negative control, Lane 3, 5-9, 11: Positive amplification of the gene.**

1 2 3 4 5 6 7 8 9 10 11


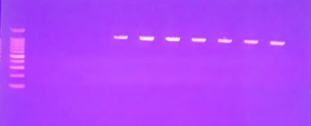


1173 bp

**Fig.S15. Agarose gel (1.5%) electrophoresis of amplified products of *CRISPR2* at ⁓ 1173 bps. Lane 1: (1500 bp) Marker, Lane 2: Negative control, Lane 5-11: Positive amplification of the gene.**


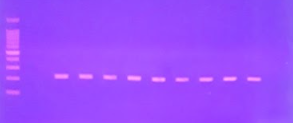


1 2 3 4 5 6 7 8 9 10 11

208 bp

**Fig.S16. Agarose gel (1.5%) electrophoresis of amplified products of *cas1* at ⁓ 208 bps. Lane 1: (1500 bp) Marker, Lane 2: Negative control, Lane 3-11: Positive amplification of the gene.**

1 2 3 4 5 6 7 8 9 10 11


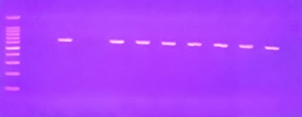


620 bp

**Fig.S17. Agarose gel (1.5%) electrophoresis of amplified products of *cas3* at ⁓ 620 bps. Lane 1: (1500 bp) Marker, Lane 2: Negative control, Lane 3, 5-11: Positive amplification of the gene.**
